# Gender differences in changes in metabolic syndrome status and its components and risk of cardiovascular disease: a longitudinal cohort study

**DOI:** 10.1186/s12933-022-01665-8

**Published:** 2022-11-02

**Authors:** Azra Ramezankhani, Fereidoun Azizi, Farzad Hadaegh

**Affiliations:** 1 Prevention of Metabolic Disorders Research Center, Research Institute for Endocrine Sciences, Shahid Beheshti University of Medical Sciences, Tehran, Iran; 2 Endocrine Research Center, Research Institute for Endocrine Sciences, Shahid Beheshti University of Medical Sciences, Tehran, Iran

**Keywords:** Metabolic syndrome, Gender, Cardiovascular, Risk

## Abstract

**Background:**

We aimed to investigate the gender difference in the association between changes in metabolic syndrome (MetS) and its components with the risk of cardiovascular disease (CVD) and coronary heart disease (CHD) among adult participants in the Tehran lipid and glucose study cohort.

**Methods:**

A total of 4624 adults (aged ≥ 30 years) who participated in two Phases 2 (2002–2005) and 3 (2005–2008) were included and followed up until 2018. Based on the status of MetS and its components in two phases, we divided participants into four groups: MetS-free, MetS-developed, MetS-recovery and MetS-stable groups, and similar categories were defined for MetS components. Multiple Cox regression models were used to estimate the adjusted hazard ratios (HRs) and 95% confidence intervals (95% CIs), and women-to-men ratios of HRs (RHRs).

**Results:**

During a median follow-up of 11.6 years, 619 CVD events (292 women) and 512 CHD events (230 women) occurred. In both genders, the MetS-stable group had the highest risk of CVD and CHD, compared with the MetS-free group, but the associations were stronger in women than men: the HR (95% CI) were (2.76, 2.00-3.82) and (3.08, 2.15–4.40) for CVD and CHD, respectively, in women, and (1.60, 1.23–2.09) and (1.74, 1.30–2.31) for men. The multivariate adjusted women-to-men RHRs were (1.72, 1.16–2.56) for CVD and (1.77, 1.14–2.73) for CHD. Only among women, the risks for CVD in MetS-recovery group (1.67, 1.06–2.63) and MetS-developed group (1.89, 1.16–3.06|) were higher than MetS-free group. For CHD, women in MetS-developed group (1.86, 1.07–3.22) had higher risk than MetS-free group. However, no evidence of gender difference was observed in these associations. Among MetS components, persistent high blood pressure (BP) conferred greater risk for CVD and CHD in women than men; the women-to-men RHRs of CVD and CHD for high BP-stable groups were 1.54 (1.05–2.26) and 1.62 (1.07–2.47), respectively. For CHD events, persistent high fasting plasma glucose was associated with greater risk in women than men with women-to-men RHRs of 1.62 (1.09–2.40).

**Conclusion:**

Change in MetS and its key components were associated with different risks for CVD events in both genders, with generally stronger associations in women than men.

**Supplementary Information:**

The online version contains supplementary material available at 10.1186/s12933-022-01665-8.

## Background

Metabolic syndrome (MetS) refers to clustering of several cardiovascular disease (CVD) risk factors including central obesity, insulin resistance, high blood pressure (BP), reduced levels of serum high-density lipoprotein cholesterol (HDL-C) and increased level of serum triglycerides (TG) [1]. Previous studies have shown that MetS increases the risk of type 2 diabetes (T2D) [2, 3], CVD [4–7], cancer [8, 9] and CV mortality [4, 7, 10]. MetS is becoming a public health problem worldwide [11]. In 2014, the global prevalence of MetS was 10.0–84.0%, being influenced by certain socio-economic and demographic factors [12]. A recent meta-analysis reported a worldwide prevalence of MetS ranging from 12.5 to 31.4% among the general adult population globally. The prevalence was significantly higher in Eastern Mediterranean Region (EMR) and Americas and increased with country’s level of income [13]. In 2016, a nationwide study in Iran showed that the prevalence of MetS was 38.3% among adults ≥ 18 years with the higher prevalence in women and in urban residents [14]. Moreover, the incidence rate of MetS among Tehranian adult women and men were reported to be more than 400 and 700 per 10,000 person years, respectively [15].

The available clinical evidence have demonstrated gender difference in the cardiovascular consequences of MetS [16] and major CVD risk factors including hypertension, diabetes and obesity [17]. A meta-analysis conducted by Gami et al. [16] has reported a stronger association between MetS and CVD in women than men. Moreover, other meta-analysis found that the presence of diabetes was associated with a 27% higher risk of stroke in women than in men [18]. The aetio-pathophysiology for this gender difference is incompletely understood, due to inadequate inclusion of women in clinical trials and a lack of pre-specified analyses of this factor [17]. However, genetic predisposition, hormonal factors [17], culture and socioeconomic factors might be important contributors [19].

Most previous published studies on the relation between MetS and CVD, have examined the association between MetS status at baseline with the incidence of CVD events [20–24]. As lifestyle modification or medical treatment can improve each MetS component, therefore, MetS status may change during the follow-up. Only a few studies have investigated the impact of changes in MetS status on CVD incidence [25, 26]; but, these studies did not explore gender differences in the relationship between changes in MetS status and CVD risk [25, 26]. Additionally, these studies have been conducted in East Asian countries, where the prevalence of overweight and obesity is generally lower than the EMR countries [27]. Therefore, in this study we aimed to assess the associations between changes in MetS status and its components with the risk of CVD events among Tehranian adults. Moreover, we compared these associations in women versus men for CVD and CHD events; because, clinical guidelines encourage investigation of the sex-specific role of biological and non-biological factors in cardiovascular health [19, 28].

## Methods

### Study population

We used data from the Tehran lipid and glucose study (TLGS), a long term, population-based prospective study that initiated in 1999 with randomly selected participants from the Tehran, capital of Iran. The TLGS was designed to investigation of the prevalence and incidence of non-communicable diseases (NCD) and their risk factors among Iranians. Details of the study design and procedure have been described elsewhere [29, 30]. Briefly, in phase 1 (1999–2002) a total of 15,005 individuals aged ≥ 3 years were recruited and relevant data were collected using the questionnaire interviews and health examinations. Measurements were then repeated triennially in phase 2 (2002–2005), phase 3 (2005–2008), phase 4 (2009–2011), phase 5 (2012–2015), and phase 6 (2015–2018). In this study, we selected data from phase 2 onward. The main reasons for this were that from phase 2, the history of CVD was accurately measured using the appropriate questionnaires [31]. Second, physical activity level (PAL) was measured based on metabolic equivalent of task value (MET) from phase 2 onward [32]. Third, in phase 2, a new sample (3550 individuals) were added to the study, that provided a larger population for study than phase 1 [31]. Accordingly, we selected 7213 participants aged ≥ 30 years in phase 2, and excluded those individuals who did not participate in phase 3 (n = 1427). Among the remaining 5786 individuals, we excluded individuals with a history of cancer (n = 33), pregnant women (n = 55), those with history of CVD (n = 445) at phases 2 and 3, and all participants who developed CVD events between phase 2 and 3 (n = 39).

**Fig. 1:**
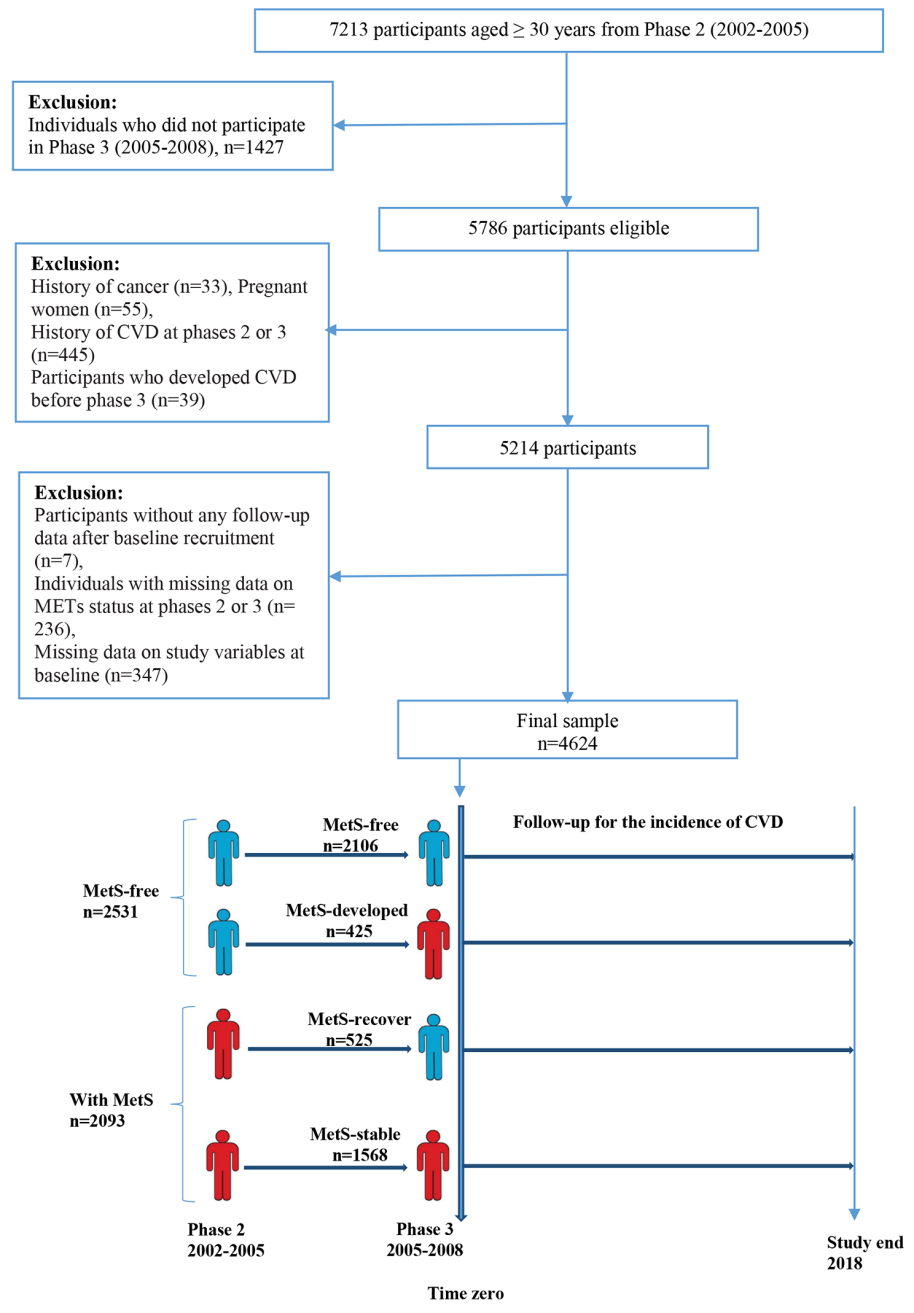
Flowchart of sample selection for the study, the TLGS study

We used data of the phases 2 and 3 to define the change in MetS status. The phase 3 was considered as the cohort entry (baseline), and information in this phase were collected and follow-up was initiated. Therefore, we excluded participants without any follow-up data after baseline recruitment (n = 7) and those individuals with missing data on MetS status at phases 2 and 3 (n = 236), and missing data on other study variables at baseline (n = 347). Finally 4624 individuals (1940 men) were included in the study and were followed up to determine the development of CVD events until the end of the study (20 March 2018) (Fig. 1). Study protocols were approved by the ethical committee of the Research Institute for Endocrine Sciences of Shahid Beheshti University of Medical Sciences, Tehran, Iran, and written informed consent was obtained from the study participants.

## Measurements

Baseline data (at phase 3) including socio-demographic information, lifestyles, medical history, and family history were collected by the face to face interview using a standard questionnaire. Family history of CVD (FH-CVD) was defined as having a female first degree relative < 65 years, or any male first degree relative < 55 years having a history of CVD event. Educational level was categorized as: less than 6 years, 6–12 years, and more than 12 years of schooling. Marital status was categorized as the single, married and widowed/divorced. Smoking status was divided into three categories: current smoker, past-smoker and never smoker. A current smoker was defined as a person who smokes cigarettes or other smoking implements daily or occasionally. Never smokers included subjects who had never smoked, and past smokers were defined as having quit smoking for at least 1 year prior to study entry. PAL was evaluated using the Modifiable Activity Questionnaire [33], and high PAL was defined as achieving a score ≥ 600 MET/week [32].

Anthropometric data including height, weight, waist circumference (WC) were measured using standard protocols [29, 30]. Body mass index (BMI) was calculated as weight in kilograms divided by height in square meters (kg/m^2^). BP was measured with a standard mercury sphygmomanometer on the right arm of seated participants after a 5-minute rest. Systolic blood pressure (SBP) and diastolic blood pressure (DBP) were calculated as the average of two measurements at 15-minutes intervals. Blood samples were taken from all participants in the morning after an overnight fast to measure biochemical indicators including fasting plasma glucose (FPG), HDL-C and TG using the enzymatic colorimetric method [30].

## Definition of MetS and study groups

MetS was defined by the presence of at least three of the following criteria [34, 35]: (1) elevated FPG (≥ 5.6 mmol/L) or use of anti-hyperglycemic agents, (2) elevated serum TG (≥ 1.7 mmol/L) or using lipid-lowering drugs, (3) reduced HDL-C (< 1.03 mmol/L for men and < 1.29 mmol/L for women), (4) elevated BP (≥ 130/85 mmHg or treatment with anti-hypertensive medications), and (5) elevated WC (≥ 95 cm according to population- and country-specific thresholds for Iranian men and women) [35]. We divided study participants into 4 groups according to the MetS status in phase 2 and 3 (Fig. 1): MetS-free (absence of MetS in both phases 2 and 3); MetS-developed (absence of MetS at phase 2 but presence of MetS at phase 3); MetS-recovery (presence of MetS at phase 2 but absence of MetS at phase 3); and MetS-stable (presence of MetS in both phases 2 and 3).

## Study outcomes

The outcomes of this study were CVD and CHD. Details of the collection of outcomes have been published previously [31]. In brief, all TLGS study participants were followed up annually for any medical events from the baseline examination until the date of first documented events. The data collected were reviewed by an expert panel to ensure the accuracy of the outcome data. The final diagnosis for each outcome was recorded using the ICD-10th edition code. In this study, CHD was defined as any definite myocardial infarction (MI) diagnosed by electrocardiogram (ECG) and biomarkers, probable MI (positive ECG findings plus cardiac symptoms and missing biomarkers or positive ECG findings plus equivocal biomarkers), unstable angina pectoris (new cardiac symptoms or changes in the symptom pattern, and positive ECG findings with normal biomarker values), and angiography proven CHD and congestive heart failure (CHF). We defined CVD as a composite of CHD plus fatal and non-fatal stroke and deaths attributable to CVD. In our study, follow-up initiated after phase 3, as the second examination, and ended on the date of the first outcome or censoring date, whichever came first. Participants were censored for non-CVD death, loss to follow-up, or the end of the study with no events occurring.

### Statistical analysis

Continuous and categorical variables are presented as mean (standard deviation (SD)) and number (percentage), respectively. For continuous variables with skewed distribution (FPG and TG), the median (interquartile range) are shown. At phases 2 and 3, we compared the characteristics between men and women. Moreover, at phase 3 (as baseline), we compared the characteristics between four groups of MetS change status in men and women. We also compared baseline characteristics between participants and non-participants. Non-participants included subjects who did not participate in phase 3 or those who had missing information on MetS status at phases 2 or 3 or who had missing data on baseline (phase 3) variables or those with no follow-up data after phase 3.

Comparisons were performed using student’s t-test and one-way analysis of variance (ANOVA) for continuous variables and chi-squared test for categorical variables. Continuous variables with skewed distribution were compared using the Mann-Whitney U test and the Kruskal-Wallis test.

For each outcome, a crude incidence rate and 95% confidence interval (CI) per 1000 person-years were calculated. Multivariable Cox proportional hazard regression model was used to test hazard ratios (HR) and 95% CI for the association between MetS and its components at baseline (phase 3) and the risk of outcomes. We also examined the association between changes in MetS and its components and the outcomes risk. Model 1 was adjusted for age (years). Model 2 was further adjusted for smoking status, PAL, education, marital status, FH-CVD, BMI and other components of MetS. To estimate the women-to-men ratios of hazard ratios (RHRs) and 95% CI, we included an interaction term of each exposure variable (i.e., baseline MetS, baseline MetS components, change in MetS and changes in MetS components) with sex in multivariable Cox models. All analyses were performed using R software (Version 4.1.2) and two-sided P < 0.05 was considered as statistically significant.

## Results

### Baseline characteristics

Of the 4624 eligible participants at baseline (phase 3), 41.9% were male and the mean age was 50.8 years. The baseline characteristics of the study population are presented in Supplementary Material 1: Table S1. The mean age of males was significantly higher than that of females. Women had significantly higher mean BMI and HDL-C; but, lower mean WC, SBP, DBP, TG and FPG than men. In addition, women were less likely to have MetS at baseline than men. The characteristics of participants at phase 2, is shown in Supplementary Material 2: Table S2. The results were similar to those observed in phase 3.

In Supplementary Material 3: Table S3, we have shown the baseline characteristics of participants and non-participants. Compared with participants, non-participants had higher mean age and SBP, and a lower mean BMI. They were more likely than participants to be smoker. Non-participants also had higher rates of low PAL, FH-CVD and use of anti-hypertensive and anti-diabetic drugs than participants.

Over 3 years, the MetS status changed for 20.5% of the total study population. Of the 2093 people with MetS at phase 2, about 25% had recovered from MetS at phase 3. In contrast, of the 2531 people without MetS at phase 2, about 17% had developed MetS by phase 3 (Fig. 1). The corresponding values for men and women are presented in Supplementary Material 4: Table S4.

Baseline characteristics of men and women by the MetS change status are shown in Tables 1 and 2, respectively. In both genders, the MetS-stable group had the highest mean age, BMI, WC, SBP, DBP, TG, and FPG, and the lowest mean HDL-C. Furthermore, in both men and women, taking lipid-lowering, anti-hypertensive and anti-diabetic drugs were significantly higher in MetS-stable groups, compared with the other groups.

**Table 1:** Baseline characteristics of men by change in MetS status, Tehran Lipid and Glucose Study

	MetS-free (n = 804)	MetS-developed (n = 215)	MetS-recovery (n = 237)	MetS-stable (n = 684)	P-value
**Continuous variable**					
Age, year	49.6 (12.9)	50.1 (12.6)	51.5 (12.2)	54.1 (12.8)	< 0.001
BMI, kg/m^2^	25.0 (3.5)	27.4 (2.8)	27.0 (3.3)	29.3 (3.7)	< 0.001
WC, cm	90.4 (9.1)	98.1 (6.4)	96.1 (8.9)	102.9 (8.2)	< 0.001
SBP, mmHg	113.8 (15.5)	121.9 (16.5)	119.5 (17.1)	128.1 (18.8)	< 0.001
DBP, mmHg	73.3 (9.1)	77.2 (9.0)	76.1 (9.7)	80.8 (10.2)	< 0.001
TG, mmol/L^*^	1.3 (0.7 )	2.0 (0.9)	1.4 (0.5)	2.3 (1.3)	< 0.001
FPG, mmol/L^*^	4.8 (0.5)	5.1 (0.8)	5.1 (0.6)	5.5 (1.4)	< 0.001
HDL-C, mmol/L	1.06 (0.24)	0.90 (0.19)	1.01 (0.19)	0.87 (0.16)	< 0.001
**Categorical variable**					
Smoking					
Current smoker	246 (30.6)	58 (27.0)	70 (29.5)	180 (26.3)	0.586
Past smoker	122 (15.2)	40 (18.6)	39 (16.5)	117 (17.1)	
never smoker	436 (54.2)	117 (54.4)	128 (54.0)	387 (56.6)	
Education					
< 6 years	173 (21.5)	42 (19.5)	55 (23.2)	209 (30.6)	0.001
6–12 years	451 (56.1)	121 (56.3)	133 (56.1)	355 (51.9)	
> 12 years	180 (22.4)	52 (24.2)	49 (20.7)	120 (17.5)	
Marital status					
Single	40 (5.0)	11 (5.1)	9 (3.8)	10 (1.5)	0.011
Married	743 (92.4)	198 (92.1)	224 (94.5)	661 (96.6)	
widowed/divorced	21 (2.6)	6 (2.8)	4 (1.7)	13 (1.9)	
Physical activity level (low)	300 (37.3)	87 (40.5)	88 (37.1)	268 (39.2)	0.772
FH-CVD (yes)	59 (7.3)	16 (7.4)	19 (8.0)	62 (9.1)	0.657
Anti-hypertensive drug use (yes)	11 (1.4)	2 (0.9)	7 (3.0)	48 (7.0)	< 0.001
Anti-diabetic drug use (yes)	5 (0.6)	8 (3.7)	11 (4.6)	93 (13.6)	< 0.001
Lipid-lowering drug use (yes)	3 (0.4)	9 (4.2)	3 (1.3)	42 (6.1)	< 0.001

The characteristics are presented at phase 3 (2005–2008) (defined as baseline). Data are shown as mean (SD) for continuous variables or number (percent) for categorical variables.

*Data are shown as median (IQR), due to skewed distribution, and comparisons were done by Kruskal-Wallis test. SBP: systolic blood pressure; DBP: diastolic blood pressure; BMI: body mass index; FPG: fasting plasma glucose; TG: Triglycerides; CVD: cardiovascular diseases; HDL-C: high-density lipoprotein cholesterol; FH-CVD: family history of CVD; MetS: metabolic syndrome; SD: standard deviation; IQR: interquartile range

**Table 2:** Baseline characteristics of women by change in MetS status, Tehran Lipid and Glucose Study

	MetS-free (n = 1302)	MetS-developed (n = 210)	MetS-recovery (n = 288)	MetS-stable (n = 884)	P-value
**Continuous variable**					
Age, year	45.4 (9.8)	51.7 (10.7)	52.3 (10.1)	56.4 10.9)	< 0.001
BMI, kg/m^2^	27.2 (3.8)	30.1 (3.9)	30.0 (4.1)	31.8 (4.7)	< 0.001
WC, cm	85.4 (10.1)	96.0 (8.3)	94.2 (9.4)	100.9 (9.6)	< 0.001
SBP, mmHg	106.6 (13.5)	121 (17.1)	115.9 (16.7)	129.5 (20.8)	< 0.001
DBP, mmHg	70.2 (8.6)	77.3 (9.5)	74.3 (9.2)	78.5 (10.5)	< 0.001
TG, mmol/L^*^	1.2 (0.7)	2.0 (0.7)	1.5 (0.6)	2.2 (1.2)	< 0.001
FPG, mmol/L^*^	4.7 (0.4)	5.1 (0.9)	4.9 (0.6)	5.7 (1.9)	< 0.001
HDL-C, mmol/L	1.22 (0.27)	1.07 (0.23)	1.15 (0.27)	1.03 (0.21)	< 0.001
**Categorical variable**					
Smoking					
Current smoker	69 (5.3)	11 (5.2)	12 (4.2)	37 (4.2)	0.351
Past smoker	24 (1.8)	1 (0.5)	5 (1.7)	24 (2.7)	
never smoker	1209 (92.9)	198 (94.3)	271 (94.1)	823 (93.1)	
Education					
< 6 years	319 (24.5)	95 (45.2)	135 (46.9)	556 (62.9)	< 0.001
6–12 years	791 (60.8)	97 (46.2)	133 (46.2)	295 (33.4)	
> 12 years	192 (14.7)	18 (8.6)	20 (6.9)	33 (3.7)	
Marital status					
Single	72 (5.5)	3 (1.4)	8 (2.8)	13 (1.5)	< 0.001
Married	1102 (84.6)	179 (85.2)	239 (83.0)	667 (75.5)	
widowed/divorced	128 (9.8)	28 (13.3)	41 (14.2)	204 (23.1)	
Physical activity level (low)	373 (28.6)	64 (30.5)	85 (29.5)	326 (36.9)	0.001
FH-CVD (yes)	138 (10.6)	27 (12.9)	28 (9.7)	94 (10.6)	0.722
Anti-hypertensive drug use (yes)	15 (1.2)	21 (10.0)	10 (3.5)	154 (17.4)	< 0.001
Anti-diabetic drug use (yes)	11 (0.8)	12 (5.7)	15 (5.2)	185 (20.9)	< 0.001
Lipid-lowering drug use (yes)	21 (1.6)	21 (10.0)	6 (2.1)	148 (16.7)	< 0.001

The characteristics are presented at phase 3 (2005–2008) (defined as baseline). Data are shown as mean (SD) for continuous variables or number (percent) for categorical variables.

* Data are shown as median (IQR), due to skewed distribution, and comparisons were done by Kruskal-Wallis test. SBP: systolic blood pressure; DBP: diastolic blood pressure; BMI: body mass index; FPG: fasting plasma glucose; TG: Triglycerides; CVD: cardiovascular diseases; HDL-C: high-density lipoprotein cholesterol; FH-CVD: family history of CVD; MetS: metabolic syndrome; SD: standard deviation; IQR: interquartile range

During a median follow-up of 11.6 years, 619 CVD events (292 women) and 512 CHD events (230 women) occurred. During the study period, 86 CV death were observed from the total number of 312 all-cause death. Crud incidence rates of CVD and CHD were 12.7 (11.7–13.7) and 10.4 (9.5–11.4) per 1000 person-years, respectively, in total population. Supplementary Material 5: Table S5, presents crude incidence rates for CVD and CHD by MetS at baseline and change in MetS status. In both genders, the baseline MetS was associated with higher incidence rate of CVD and CHD, compared with MetS-free group. Also, incidence rate of CVD and CHD was highest in the MetS-stable group, compared with the MetS-free group.

## Baseline MetS and its components and the risk of outcomes

In Supplementary Material 6: Figure S1, we have shown the association of baseline MetS with the risk of developing CVD and CHD. Moreover, in Supplementary Material 7: Table S6 and Supplementary Material 8: Table S7, association between MetS components at baseline and CVD and CHD risk has been presented. The presence of MetS at baseline was significantly associated with an increased risk of CVD and CHD, in both genders even after controlling for age, smoking status, PAL, education, marital status, FH-CVD and BMI. However, the associations were stronger in women than men; the multivariable-adjusted women-to-men RHRs were 1.51 (1.07–2.12) for CVD and 1.65 (1.13–2.40) for CHD (Supplementary Material 6: Figure S1).

Among the individual components of MetS at baseline, elevated BP in multivariate adjusted models was significantly associated with CVD and CHD in both genders, with a higher risk for women than men (the women-to-men RHRs for elevated BP were 1.42 (1.03–1.97) and 1.42 (1.01–2.04) for CVD and CHD, respectively). Furthermore, in both genders, high FPG were significantly associated with the risk of CVD and CHD even after controlling for confounders; however, gender difference in these associations were found only for CHD (women-to-men RHRs: 1.63 (1.14–2.33)). High TG was associated with increased risk of CVD and CHD in both genders. However, after controlling for confounders, the associations remained significant only among women for CHD. But, no evidence of gender difference was found in this association (Supplementary Material 7: Table S6 and Supplementary Material 8: Table S7).

## Changes in MetS and its components and the risk of outcomes

**Fig. 2:**
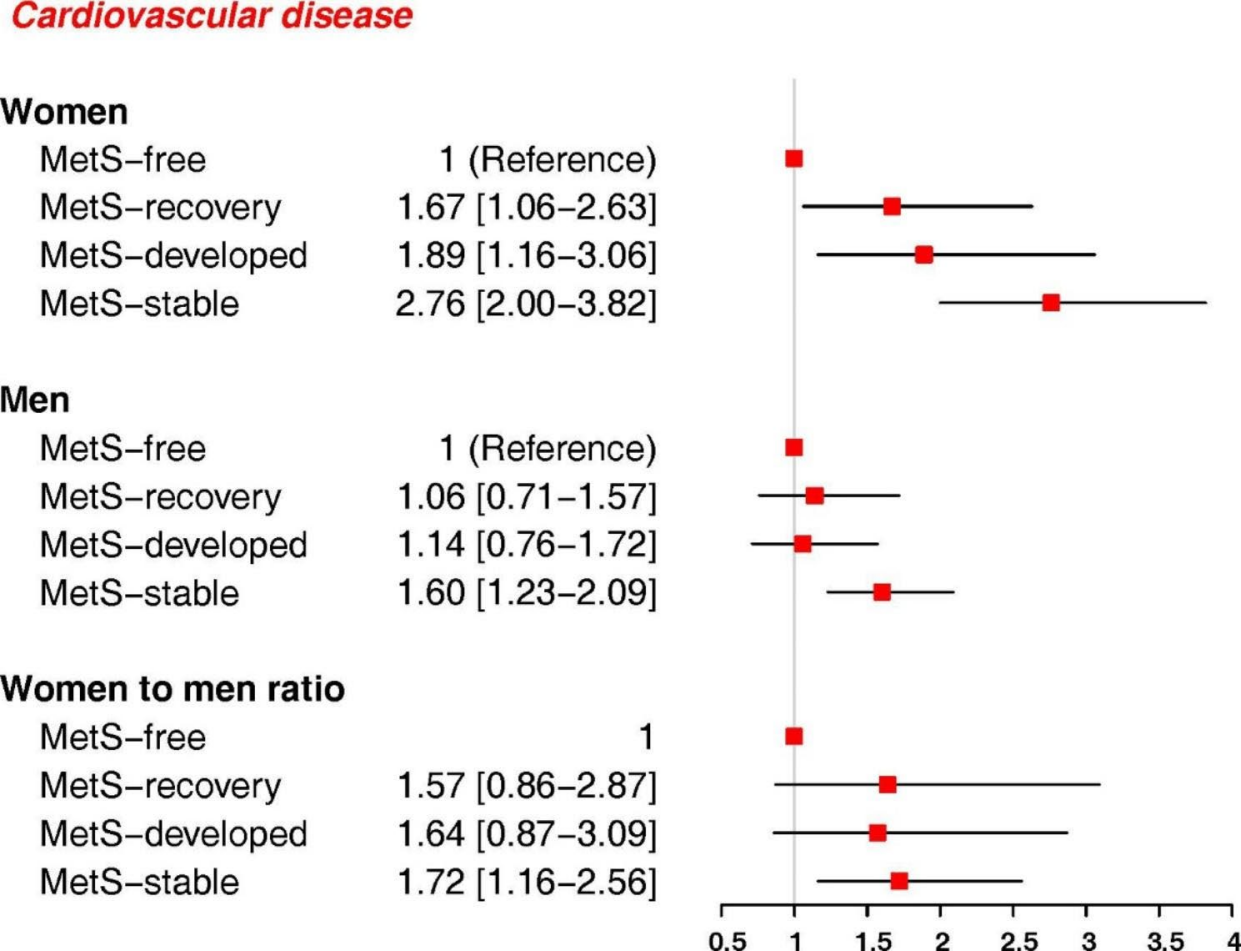
Association of change in MetS status with the risk of CVD, Tehran Lipid and Glucose Study. HR: hazard ratio; MetS: metabolic syndrome; CVD: cardiovascular disease; HR was estimated using COX regression model adjusted for age, smoking status, physical activity level, education, marital status, family history of CVD, body mass index

**Fig. 3:**
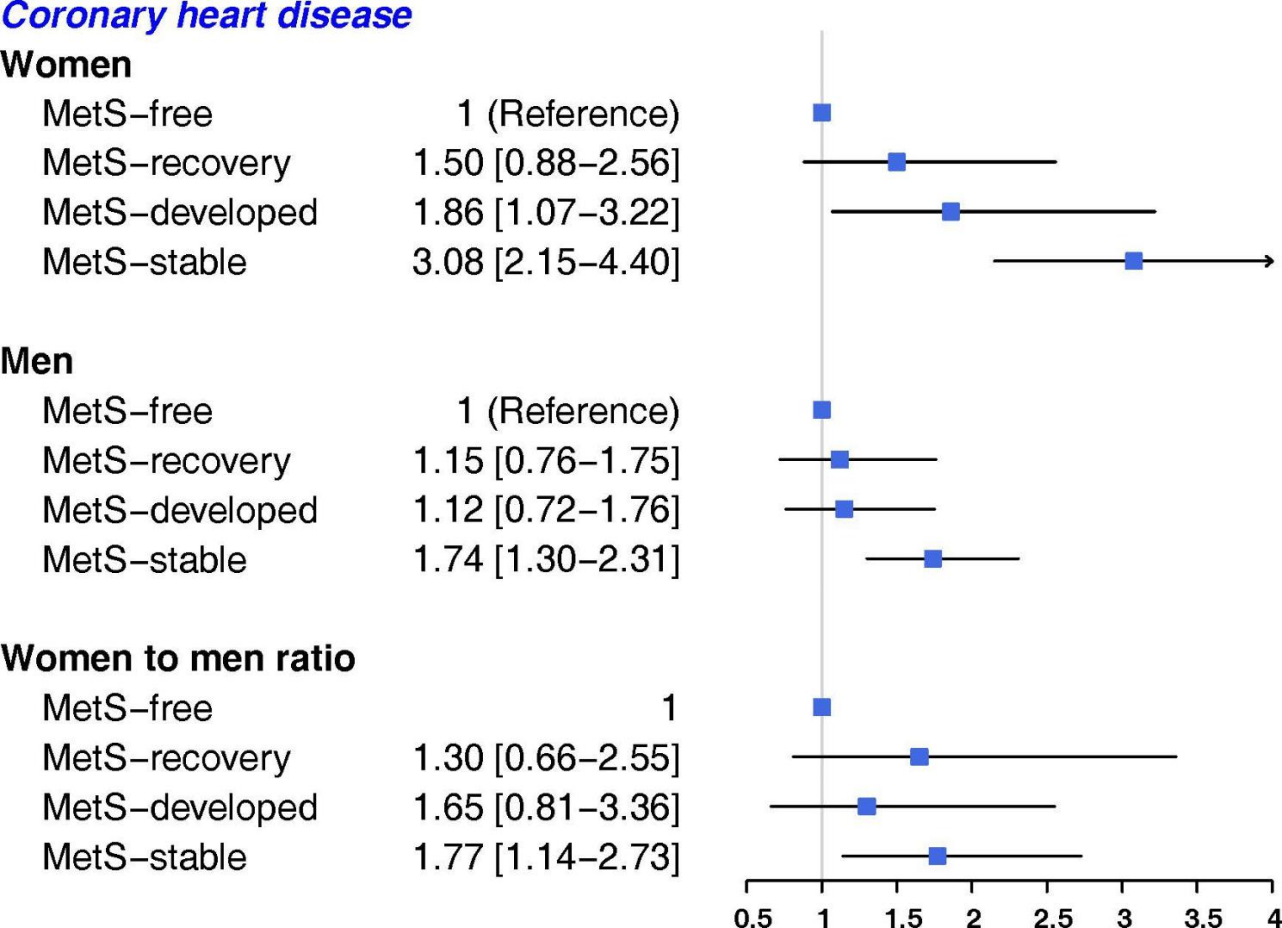
Association of change in MetS status with the risk of CHD, Tehran Lipid and Glucose Study. HR: hazard ratio; MetS: metabolic syndrome; CHD: Coronary heart disease; HR was estimated using COX regression model adjusted for age, smoking status, physical activity level, education, marital status, family history of CVD, body mass index

Figures 2 and 3 show that in both genders, after controlling for age, smoking status, PAL, education, marital status, FH-CVD and BMI, the MetS-stable group had the highest risk of CVD (Fig. 2) and CHD (Fig. 3), compared with the MetS-free group, with the associations being stronger in women than in men (women-to-men RHRs: 1.72 (1.16–2.56) for CVD and 1.77 (1.14–2.73) for CHD). The MetS-developed and MetS-recovery groups had a higher risk of CVD and CHD than the MetS-free group, only among women. However, the risks were lower than those with MetS-stable groups. No evidence of gender difference was observed in these associations.

Among the components of MetS, persistent status of all components, except for persistent low HDL-C were significantly associated with an increased risk for CVD and CHD in women. However, after adjusting for confounders, the effect disappeared for persistent high WC (for CVD). In men, persistent status of all components was significantly associated with increased risk for CVD and CHD. After controlling for confounders, the associations were significant only for the persistent status of high FPG and high BP for both CVD and CHD. The HRs of CVD and CHD for high BP-stable groups were higher in women than men; the women-to-men RHRs were 1.54 (1.05–2.26) for CVD and 1.62 (1.07–2.47) for CHD. Also, persistent high FPG was associated with a higher risk of CHD in women than in men, with a women-to-men RHRs of 1.62 (1.09–2.40) (Tables 3 and 4).

**Table 3:** Association of changes in MetS components with the risk of CVD, Tehran Lipid and Glucose Study

	Women (n = 2684)					Men (n = 1940)				
			**Model 1**	**Model 2**				**Model 1**	**Model 2**	
**Parameter**	**Events/n**	**Incidence** **Rate per 1000** **Person-Years**	**HR (95% CI)**	**HR (95% CI)**		**Events/n**	**Incidence** **Rate per 1000** **Person-Years**	**HR (95% CI)**	**HR (95% CI)**	* Women-to-men RHR
**High WC**										
High WC-free	82/1288	3.8 (3.1–4.7)	Reference	Reference		100/729	8.6 (7.0-10.5)	Reference	Reference	**-**
High WC-recovery	26/224	6.9 (4.7–10.2)	1.30 (0.83–2.02)	1.16 (0.74–1.82)		18/82	14.4 (9.0-22.8)	1.69 (1.03–2.80)	1.54 (0.93–2.56)	0.75 (0.38–1.47)
High WC-developed	20/223	5.3 (3.4–8.3)	1.13 (0.69–1.84)	0.98 (0.59–1.61)		30/202	9.3 (6.5–13.3)	1.09 (0.72–1.64)	0.99 (0.65–1.51)	0.98 (0.51–1.86)
High WC-stable	164/949	10.6 (9.1–12.4)	1.74 (1.33–2.28)	1.35 (0.98–1.87)		179/927	12.1 (10.5–14.1)	1.31 (1.02–1.67)	1.07 (0.80–1.43)	1.26 (0.87–1.82)
**High FPG**										
High FPG-free	125/1796	4.1 (3.4–4.9)	Reference	Reference		180/1275	8.8 (7.6–10.1)	Reference	Reference	-
High FPG-recovery	17/166	6.2 (3.9–10.1)	1.11 (0.67–1.84)	0.95 (0.57–1.58)		20/152	7.9 (5.1–12.3)	0.68 (0.43–1.08)	0.65 (0.41–1.04)	1.44 (0.72–2.87)
High FPG-developed	17/156	6.4 (4.0-10.4)	1.30 (0.78–2.15)	1.15 (0.69–1.91)		29/163	11.2 (7.7–16.1)	1.05 (0.71–1.55)	0.98 (0.66–1.45)	1.16 (0.61–2.22)
High FPG-stable	133/566	15.0 (12.6–17.8)	2.39 (1.86–3.06)	1.89 (1.46–2.45)		98/350	18.8 (15.4–23.0)	1.55 (1.21–1.99)	1.41 (1.09–1.81)	1.34 (0.94–1.91)
**High TG**										
High TG-free	62/1107	3.3 (2.5–4.2)	Reference	Reference		114/706	10.2 (8.4–12.2)	Reference	Reference	**-**
High TG-recovery	39/288	8.2 (6.0-11.3)	1.92 (1.28–2.86)	1.76 (1.18–2.64)		37/244	9.5 (6.9–13.1)	1.20 (0.83–1.74)	1.10 (0.75–1.60)	1.60 (0.92–2.78)
High TG-developed	33/319	6.2 (4.4–8.8)	1.51 (0.99–2.30)	1.44 (0.94–2.21)		32/199	10.0 (7.1–14.2)	1.12 (0.76–1.66)	1.02 (0.69–1.52)	1.41 (0.79–2.51)
High TG-stable	158/970	10.1 (8.6–11.7)	2.09 (1.55–2.81)	1.60 (1.17–2.19)		144/791	11.5 (9.7–13.5)	1.37 (1.07–1.76)	1.17 (0.90–1.52)	1.37 (0.92–2.02)
**Low HDL**										
Low HDL-free	32/307	6.3 (4.4–8.9)	Reference	Reference		50/322	9.8 (7.4–13.0)	Reference	Reference	**-**
Low HDL-recovery	38/397	5.7 (4.1–7.8)	0.98 (0.61–1.56)	0.96 (0.60–1.55)		51/348	9.1 (6.9–11.9)	1.11 (0.75–1.64)	1.10 (0.74–1.62)	0.87 (0.47–1.62)
Low HDL-developed	14/149	5.8 (3.4–9.8)	0.93 (0.50–1.75)	0.89 (0.47–1.67)		23/129	11.6 (7.7–17.5)	1.37 (0.84–2.25)	1.25 (0.76–2.06)	0.70 (0.31–1.57)
Low HDL-stable	208/1831	6.8 (6.0-7.8)	1.14 (0.79–1.66)	0.94 (0.64–1.38)		203/1141	11.2 (9.7–12.8)	1.42 (1.04–1.93)	1.17 (0.85–1.62)	0.80 (0.49–1.30)
**High BP**										
High BP-free	75/1605	2.7 (2.1–3.4)	Reference	Reference		117/1077	6.6 (5.5–7.9)	Reference	Reference	-
High BP-recovery	40/301	8.1 (6.0-11.1)	1.92 (1.30–2.83)	1.77 (1.19–2.61)		39/201	12.6 (9.2–17.2)	1.53 (1.06–2.20)	1.45 (1.01–2.09)	1.21 (0.71–2.06)
High BP-developed	32/222	8.8 (6.2–12.4)	2.25 (1.48–3.41)	1.98 (1.30–3.01)		45/231	12.2 (9.1–16.4)	1.29 (0.91–1.83)	1.34 (0.94–1.90)	1.47 (0.86–2.53)
High BP-stable	145/556	16.8 (14.3–19.8)	3.15 (2.35–4.23)	2.55 (1.88–3.45)		126/431	19.6 (16.0-23.3)	1.72 (1.32–2.23)	1.65 (1.26–2.16)	1.54 (1.05–2.26)

Model 1: Adjusted for age, Model 2: Adjusted for age, smoking status, physical activity level, education, marital status, family history of CVD, body mass index + other components of MetS.

*Women to men RHR: The value shows women-to-men relative hazard ratio for each parameter obtained in model 2. MetS: metabolic syndrome; CVD: cardiovascular diseases; CI: confidence interval; HR: hazard ratio; RHR: ratio of hazard ratios; BP: blood pressure; FPG: fasting plasma glucose; TG: Triglycerides; HDL-C: high-density lipoprotein cholesterol; WC; waist circumference

**Table 4:** Association of changes in MetS components with the risk of CHD, Tehran Lipid and Glucose Study

	Women (n = 2684)					Men (n = 1940)				
			**Model 1**	**Model 2**				**Model 1**	**Model 2**	
**Parameter**	**Events/n**	**Incidence** **Rate per 1000** **Person-Years**	**HR (95% CI)**	**HR (95% CI)**		**Events/n**	**Incidence** **Rate per 1000** **Person-Years**	**HR (95% CI)**	**HR (95% CI)**	* Women-to-men RHR
**High WC**										
High WC-free	67/1288	3.1 (2.4–3.9)	Reference	Reference		85/729	7.3 (5.9-9.0)	Reference	Reference	**-**
High WC-recovery	19/224	5.0 (3.2–7.9)	1.20 (0.72-2.00)	1.11 (0.66–1.87)		17/82	13.4 (8.3–21.6)	1.87 (1.11–3.14)	1.73 (1.03–2.92)	0.64 (0.30–1.33)
High WC-developed	18/223	4.8 (3.0-7.6)	1.29 (0.76–2.17)	1.19 (0.69–2.03)		24/202	7.4 (4.9–11.1)	1.03 (0.65–1.62)	0.96 (0.60–1.52)	1.23 (0.61–2.47)
High WC-stable	126/949	8.1 (6.8–9.6)	1.71 (1.27–2.31)	1.48 (1.03–2.12)		156/927	10.5 (9.0-12.3)	1.35 (1.04–1.76)	1.20 (0.88–1.65)	1.23 (0.82–1.83)
**High FPG**										
High FPG-free	92/1796	3.0 (2.4–3.7)	Reference	Reference		159/1275	7.7 (6.6-9.0)	Reference	Reference	**-**
High FPG-recovery	13/166	4.7 (2.7–8.2)	1.19 (0.66–2.12)	1.00 (0.56–1.79)		18/152	7.1 (4.4–11.3)	0.72 (0.44–1.17)	0.69 (0.42–1.13)	1.44 (0.67–3.08)
High FPG-developed	17/156	6.4 (4.0-10.4)	1.82 (1.08–3.06)	1.61 (0.95–2.71)		23/163	8.8 (5.8–13.2)	0.97 (0.62–1.50)	0.91 (0.59–1.41)	1.76 (0.89–3.47)
High FPG-stable	108/566	12.1 (10.0-14.6)	2.76 (2.08–3.66)	2.20 (1.65–2.95)		82/350	15.5 (12.5–19.3)	1.50 (1.14–1.96)	1.36 (1.03–1.79)	1.62 (1.09–2.40)
**High TG**										
High TG-free	46/1107	2.4 (1.8–3.2)	Reference	Reference		88/706	7.8 (6.3–9.6)	Reference	Reference	**-**
High TG-recovery	28/288	5.8 (4.1–8.5)	1.88 (1.18–3.01)	1.70 (1.06–2.74)		36/244	9.2 (6.6–12.8)	1.46 (0.99–2.16)	1.35 (0.91–2.01)	1.26 (0.68–2.32)
High TG-developed	27/319	5.1 (3.4–7.4)	1.71 (1.06–2.75)	1.57 (0.97–2.54)		30/199	9.4 (6.6–13.5)	1.36 (0.90–2.06)	1.23 (0.81–1.88)	1.27 (0.67–2.40)
High TG-stable	129/970	8.1 (6.8–9.7)	2.38 (1.69–3.34)	1.80 (1.26–2.56)		128/791	10.1 (8.5–12.1)	1.54 (1.18–2.03)	1.31 (0.98–1.75)	1.37 (0.88–2.13)
**Low HDL**										
Low HDL-free	23/307	4.5 (3.0-6.8)	Reference	Reference		39/322	7.6 (5.6–10.5)	Reference	Reference	**-**
Low HDL-recovery	26/397	3.8 (2.6–5.7)	0.91 (0.52–1.59)	1.13 (0.73–1.75)		43/348	7.6 (5.6–10.3)	1.17 (0.76–1.81)	0.88 (0.50–1.54)	0.77 (0.37–1.57)
Low HDL-developed	13/149	5.3 (3.1–9.2)	1.19 (0.60–2.35)	1.17 (0.67–2.06)		18/129	8.9 (5.6–14.2)	1.33 (0.76–2.32)	1.09 (0.55–2.16)	0.93 (0.38–2.23)
Low HDL-stable	168/1831	5.5 (4.7–6.4)	1.27 (0.82–1.96)	1.27 (0.89–1.82)		182/1141	10.0 (8.6–11.5)	1.59 (1.12–2.24)	1.00 (0.64–1.56)	0.78 (0.44–1.37)
**High BP**										
High BP-free	66/1605	2.4 (1.9-3.0)	Reference	Reference		108/1077	6.1 (5.1–7.4)	Reference	Reference	-
High BP-recovery	29/301	5.8 (4.1–8.4)	1.65 (1.06–2.56)	1.52 (0.97–2.37)		33/201	10.5 (7.5–14.9)	1.43 (0.96–2.11)	1.34 (0.91–1.99)	1.12 (0.62–2.03)
High BP-developed	21/222	5.7 (3.7–8.7)	1.72 (1.05–2.82)	1.50 (0.91–2.47)		41/231	11.1 (8.2–15.1)	1.34 (0.93–1.93)	1.41 (0.98–2.04)	1.06 (0.57–1.95)
High BP-stable	114/556	13.1 (10.9–15.8)	3.02 (2.19–4.16)	2.43 (1.75–3.39)		100/431	15.3 (12.6–18.7)	1.54 (1.16–2.05)	1.49 (1.11-2.00)	1.62 (1.07–2.47)

Model 1: Adjusted for age, Model 2: Adjusted for age, smoking status, physical activity level, education, marital status, family history of CVD, body mass index + other components of MetS.

*Women to men RHR: The value shows women-to-men relative hazard ratio for each parameter obtained in model 2. MetS: metabolic syndrome; CVD: cardiovascular diseases; CI: confidence interval; HR: hazard ratio; RHR: ratio of hazard ratios; BP: blood pressure; FPG: fasting plasma glucose; TG: Triglycerides; HDL-C: high-density lipoprotein cholesterol; WC; waist circumference

## Discussion

In this prospective cohort study of Iranian population with more than 4500 individuals, we found that there was an independent association between MetS and increased risk of CVD and CHD in both genders, with more than 50% excess risks in women than men. In addition, we found that outcomes’ risk changed significantly according to the changes in MetS status. After controlling for confounders, those with stable MetS had the highest risk of CVD and CHD, compared with persons who remained free from MetS, in both genders; but, the risks were about 70% greater in women than men.

### MetS and its components at baseline

Our findings add to previous studies on the association of MetS and its components at baseline and the risk of CVD, including a number of systematic reviews and meta-analysis of the available literature [16, 36–38]. A systematic review study assessed sex-specific association between MetS and its components with development of CVD and showed that MetS and some of its components including diabetes and hypertension conferred a greater risk for CVD in women than men, but direct statistical comparisons between two genders were not presented [37]. The results of a meta-analysis of 37 longitudinal studies [16] showed that in total population, MetS had a relative risk (RR) of 1.78 for CVD, with the stronger association in women than in men (RR 2.63 vs. 1.98).

In line with most previous related studies [16, 37, 38], we found that MetS and its two components including high FPG and high BP were associated with increased risk of CVD and CHD, in both genders. However, the magnitude of excess risks was greater in women than in men. The gender difference in the relation between MetS and CVD can, at least in part, be attributed to the gender differences in dysglycemia, body fat, adipocyte biology, and the hormonal control of body weight [39–41]. Alternative explanations for gender differences include estrogen deficiency and dysregulated lipid metabolism in postmenopausal women [42, 43]. Moreover, we have previously shown that women experienced more metabolic disturbances (i.e., a faster adverse change in WC, SBP, DBP, and HDL-C) than men before onset of hypertension [44] and diabetes [45]. Therefore, the greater exposure of women to metabolic risk factors before diabetes and hypertension incidence may help to explain the stronger impact of high BP and FPG for CVD events in women than men [46]. Finally, it has been shown that guideline-recommended risk factor control is poorer in dysglycemic women than men and this may contribute to the worse prognosis in women with dysglycaemia and coronary artery disease (CAD) [47].

### Change in MetS and its components

Most of the previous studies that investigated the association between MetS and CVD were based on the presence of MetS at baseline [16, 37, 38]. Very few studies have considered the reversible and changeable status of MetS during follow-up [25, 26]. Using the Korean national health information database, Park et al. [25] divided study population into four groups based on the change in MetS status. During a follow-up period of 3.5 years, they found a higher risk for major adverse cardiovascular events among individuals who developed MetS, compared with those who were free of MetS. However, individuals who recovered from MetS had lower risk of events than those individuals with stable MetS. In other study on 31,481 Chinese population aged 18–98 years [26], the similar results were found for CVD and all-cause mortality. Compared with those free of MetS, individuals who developed MetS, had increased risks of CVD, MI, stroke and heart failure; however, people who recovered from MetS, had decreased risks for those outcomes, compared with the group with stable MetS. In both above mentioned studies, people with stable MetS had the highest risk for outcomes, compared with those who remained free of MetS.

Our findings about relation between change of MetS status and CVD incidence are generally consistent with those reported in the East Asian cohorts [25, 26]. We further found that persistent MetS conferred 70% higher risk in women than in men. Also we found that among women, the risk of CVD and CHD in MetS-recovery group was higher than MetS-free group, but lower than those with persistent MetS. These findings suggest that MetS is still a risk factor for CVD and CHD, even if there is a recovery of it. This phenomenon might be correlated with “metabolic memory”, a phenomenon that refers to effects of prior hyperglycemia that are ‘memorized’ by vascular cells or tissues over time and leads to increased risk for CVD, despite attainment of glycaemic control [48, 49]. Although the exact mechanism for this phenomenon is unclear, epigenetic mechanisms are thought to be involved [50]. However, lower risk of CVD and CHD in MetS-recovery group, compared with MetS-stable group, suggest that lifestyle interventions on MetS might decrease the incidences of consequent long-term CVD and CHD events [25, 26]. Additionally, gender difference in the impact of persistent MetS on risk of CVD and CHD show that lifestyle intervention programs for the MetS should consider gender differences in physiological and behavioural factors [41, 43, 51].

We further showed that alterations in some MetS components altered risks for CVD and CHD in both genders; however, the number of MetS components that affected CVD risk was higher in women than men. For example, among women, persistence of four MetS components including high WC, FPG, TG and BP was associated with highest risk of CHD, compared with remaining free of these components. Whereas, among men only persistent high FPG was associated with highest CHD risk, compared with men who remained free of high FPG. Second, the strength of association between alterations in MetS components with CVD and CHD risk was greater in women than men. For example, stable high FPG and high BP status conferred more than 60% higher risk in women than men for CHD.

In addition, improving the high BP had a greater impact on reducing the risk of CVD and CHD in women than in men; compared with individuals with stable high BP as reference group, women who recovered from high BP, had 30% lower risk for CVD and CHD [HR: 0.69 (0.49–0.99)], however, such decrease was not statistically significant in men [0.88 (0.61–1.26)] (data not shown).

Generally, our findings are consistence with current evidence regarding gender difference in consequence of CVD risk factors. It is widely accepted that women with T2D have a higher excess risk for CVD than their male counterparts. For instance, the left ventricle is subject to maladaptive changes with worsening of glucose tolerance, especially in women with newly diagnosed T2D [52]. Moreover, it has been shown that in patients with T2D, higher TG and TG/HDL ratio and lower HDL-C were independently associated with increased all-cause mortality, with a modifying effect of gender for TG and HDL-C [53], and a high triglyceride-glucose (TyG) index was significantly associated with subclinical atherosclerosis and gender disparity even in non-diabetic patients [54]. The impact of MetS components on mortality in older people presents also gender differences, with low HDL-C, hyperglycemia, and elevated BP being more strongly associated to all-cause and CVD mortality in women [55].

Taken together, the results of this study and past findings [25, 26] encourages clinicians and health care providers to pay more attention to the gender differences and also to the history of MetS even in persons who are currently free from MetS.

### Strengths and limitations

The main strengths of this study include its prospective cohort design with large sample size, and standardized measurements of metabolic markers including anthropometrics and laboratory data. Second, we used data with a relatively long follow-up period to comprehensively examine the associations of CVD events with changes in the MetS and its components, as well as gender differences in these associations, in trying to overcome limitations of previous related studies [25, 26]. To our knowledge, this study is the first to examine the relation between changes of MetS and its components with CVD events in EMR region. Some limitations in our study should be acknowledged. First, individuals with MetS at the baseline might have long exposure to MetS before entrance into the study, therefore, the effects of MetS-recovery and MetS-stable on CVD risk might be misestimated. Second, residual confounding by dietary intakes, social or clinical factors is also plausible. Finally, data were from a population of district 13 of Tehran, suggesting that our findings may not necessarily be generalizable to the rural area of Iran or other ethnic populations.

## Conclusion

Results of our study reaffirmed that MetS and its key components including high BP and FPG increase the risk of CVD events in both genders, with higher risk among women than men. We further showed that the change in MetS and its key components was closely associated with different risks for CVD in both genders, with generally stronger association in women than men. Our nationwide results have important implications in screening and prevention programs of CVD in each gender, and suggest that lifestyle intervention or pharmacological therapy may have favorable effect on the MetS, and particular attention should be paid to improving high BP and high FPG, especially among women.

## Electronic supplementary material

Below is the link to the electronic supplementary material.

Supplementary Material 1 Table S1. Baseline characteristics of the study population by sex, Tehran Lipid and Glucose Study

Supplementary Material 2 Table S2. The characteristics of the study population by sex at phase 2 (2002-2005), Tehran Lipid and Glucose Study

Supplementary Material 3 Table S3. Baseline characteristics of the participants and non-participant, Tehran Lipid and Glucose Study

Supplementary Material 4 Table S4: Study population stratified by the metabolic syndrome status at Phase 2 and Phase 3

Supplementary Material 5 Table S5: Incidence rates for CVD and CHD according to the baseline MetS* and change in MetS status, Tehran Lipid and Glucose Study

Supplementary Material 6 Figure S1: Association of MetS at baseline with the risk of CVD (left) and CHD (right), Tehran Lipid and Glucose Study. HR: hazard ratio; MetS: metabolic syndrome; CVD: Cardiovascular disease; CHD: Coronary heart disease. HR was estimated using COX regression model adjusted for age, smoking status, physical activity level, education, marital status, family history of CVD, body mass index

Supplementary Material 7 Table S6: Association of MetS components at *baseline with the risk of CVD, Tehran Lipid and Glucose Study

Supplementary Material 8 Table S7: Association of MetS components at *baseline with the risk of CHD, Tehran Lipid and Glucose Study

## Data Availability

All datasets generated and analyzed during the current study are available from the corresponding author upon reasonable request.

## List of abbreviations

**ANOVA**: Analysis of variance
**BMI**: Body mass index
**BP**: Blood pressure
**CAD**: Coronary artery disease
**CHD**: Coronary heart disease
**CHF**: Congestive heart failure
**CIs**: Confidence intervals
**CVD**: Cardiovascular disease
**DBP**: Diastolic blood pressure
**ECG**: Electrocardiogram
**EMR**: Eastern Mediterranean Region
**FH-CVD**: Family history of CVD
**FPG**: Fasting plasma glucose
**HDL-C**: High-density lipoprotein cholesterol
**HRs**: Hazard ratios
**MET**: Metabolic equivalent task minutes)/week
**MetS**: Metabolic syndrome
**MI**: Myocardial infarction
**NCD**: Non-communicable diseases
**PAL**: Physical activity level
**RR**: Relative risk
**SBP**: Systolic blood pressure
**SD**: Standard deviation
**T2D**: Type 2 diabetes
**TG**: Triglycerides
**TLGS**: Tehran lipid and glucose study
**TyG**: Triglyceride glucose
**WC**: Waist circumference
