# Supplementary material for: Gender differences in changes in metabolic syndrome status and its components and risk of cardiovascular disease: a longitudinal cohort study
Source: Cardiovasc Diabetol. 2022 Nov 2;21:227. doi: 10.1186/s12933-022-01665-8 (PMC9632145; doi:10.1186/s12933-022-01665-8)
Supplement: Supplementary file 1 — Supplementary Material 1 Table S1. Baseline characteristics of the study population by sex, Tehran Lipid and Glucose Study [file 12933_2022_1665_MOESM1_ESM.docx]

| **Table S1.** Baseline characteristics of the study population by sex, Tehran Lipid and Glucose Study | | | |
| --- | --- | --- | --- |
|  | **Men**  **(n=1940)** | **Women**  **(n=2684)** | **P-value** |
| **Continuous variable** |  |  |  |
| Age, year | 51.4 (12.9) | 50.2 (11.4) | 0.001 |
| BMI, kg/m^2^ | 27.1 (3.9) | 29.3 (4.6) | <0.001 |
| WC, cm | 96.3 (10.1) | 92.3 (12.0) | <0.001 |
| SBP, mmHg | 120.4 (18.1) | 116.3 (19.7) | <0.001 |
| DBP, mmHg | 76.7 (10.1) | 73.9 (10.1) | <0.001 |
| TG, mmol/L^*^ | 1.99 (1.20) | 1.82 (1.08) | <0.001 |
| FPG, mmol/L^*^ | 5.1 (0.7) | 4.9 (0.8) | <0.001 |
| HDL-C, mmol/L | 0.97 (0.22) | 1.14 (0.26) | <0.001 |
|  |  |  |  |
| **Categorical variable** |  |  |  |
| Smoking |  |  |  |
| Current smoker | 554 (28.6) | 129 (4.8) | <0.001 |
| Past smoker | 318 (16.4) | 54 (2.0) |  |
| never smoker | 1068 (55.1) | 2501 (93.2) |  |
| Education |  |  |  |
| < 6 years | 479 (24.7) | 1105 (41.2) | <0.001 |
| 6-12 years | 1060 (54.6) | 1316 (49.0) |  |
| > 12 years | 401 (20.7) | 263 (9.8) |  |
| Marital status |  |  |  |
| Single | 70 (3.6) | 96 (3.6) | <0.001 |
| Married | 1826 (94.1) | 2187 (81.5) |  |
| widowed/divorced | 44 (2.3) | 401 (14.9) |  |
| Physical activity level (low) | 743 (38.3) | 848 (31.6) | <0.001 |
| FH-CVD (yes) | 156 (8.0) | 287 (10.7) | 0.003 |
| Anti-hypertensive drug use (yes) | 68 (3.5) | 200 (7.5) | <0.001 |
| Anti-diabetic drug use (yes) | 117 (6.0) | 223 (8.3) | 0.004 |
| Lipid-lowering drug use (yes) | 57 (2.9) | 196 (7.3) | <0.001 |
| MetS | 899 (46.3) | 1094 (40.8) | <0.001 |
| The characteristics are presented at phase 3 (2005-2008) (defined as baseline).  Data are shown as mean (SD) for continuous variables or number (percent) for categorical variables.  * Data are shown as median (IQR), due to skewed distribution, and comparisons were done by Mann–Whitney U test.  **SBP**: systolic blood pressure; **DBP**: diastolic blood pressure; **BMI**: body mass index; **FPG**: fasting plasma glucose; **TG**: Triglycerides; **CVD**: cardiovascular diseases; **HDL-C:** high-density lipoprotein cholesterol; **FH-CVD**: family history of CVD; **MetS:** metabolic syndrome; **SD**: standard deviation; **IQR**: interquartile range | | | |
