# Supplementary material for: Gender differences in changes in metabolic syndrome status and its components and risk of cardiovascular disease: a longitudinal cohort study
Source: Cardiovasc Diabetol. 2022 Nov 2;21:227. doi: 10.1186/s12933-022-01665-8 (PMC9632145; doi:10.1186/s12933-022-01665-8)
Supplement: Supplementary file 2 — Supplementary Material 2 Table S2. The characteristics of the study population by sex at phase 2 (2002-2005), Tehran Lipid and Glucose Study [file 12933_2022_1665_MOESM2_ESM.docx]

| **Table S2.** The characteristics of the study population by sex at phase 2 (2002-2005), Tehran Lipid and Glucose Study | | | |
| --- | --- | --- | --- |
|  | **Men**  **(n=1940)** | **Women**  **(n=2684)** | **P-value** |
| **Continuous variable** |  |  |  |
| Age, year | 48.5 (12.9) | 47.4 (11.4) | 0.002 |
| BMI, kg/m^2^ | 26.7 (3.9) | 29.1 (4.6) | <0.001 |
| WC, cm | 94.7 (10.3) | 92.5 (11.6) | <0.001 |
| SBP, mmHg | 119.5 (17.4) | 117.3 (19.1) | <0.001 |
| DBP, mmHg | 76.3 (10.7) | 75.6 (10.2) | 0.015 |
| TG, mmol/L^*^ | 1.8 (1.3) | 1.6 (1.1) | <0.001 |
| FPG, mmol/L^*^ | 5.1 (0.7) | 5.0 (0.8) | <0.001 |
| HDL-C, mmol/L | 0.9 (0.3) | 1.0 (0.3) | <0.001 |
|  |  |  |  |
| **Categorical variable** |  |  |  |
| Smoking |  |  |  |
| Current smoker | 600 (31.1) | 155 (5.8) | <0.001 |
| Past smoker | 326 (16.9) | 62 (2.3) |  |
| never smoker | 1004 (52.0) | 2464 (91.9) |  |
| Education |  |  |  |
| < 6 years | 473 (24.4) | 1097 (40.9) | <0.001 |
| 6-12 years | 1079 (55.7) | 1331 (49.6) |  |
| > 12 years | 386 (19.9) | 254 (9.5) |  |
| Marital status |  |  |  |
| Single | 87 (4.5) | 106 (3.9) | <0.001 |
| Married | 1820 (93.9) | 2238 (83.4) |  |
| widowed/divorced | 31 (1.6) | 341 (12.7) |  |
| Physical activity level (low) | 856 (45.4) | 803 (30.1) | <0.001 |
| FH-CVD (yes) | 223 (11.5) | 229 (8.5) | <0.001 |
| Anti-hypertensive drug use (yes) | 79 (4.1) | 299 (11.1) | <0.001 |
| Anti-diabetic drug use (yes) | 77 (4.0) | 161 (6.0) | 0.001 |
| Lipid-lowering drug use (yes) | 42 (2.2) | 123 (4.6) | <0.001 |
| MetS | 921 (47.5) | 1172 (43.6) | 0.005 |
| The characteristics are presented at phase 2 (2002-2005).  Data are shown as mean (SD) for continuous variables or number (percent) for categorical variables.  * Data are shown as median (IQR), due to skewed distribution, and comparisons were done by Mann–Whitney U test.  **SBP**: systolic blood pressure; **DBP**: diastolic blood pressure; **BMI**: body mass index; **FPG**: fasting plasma glucose; **TG**: Triglycerides; **CVD**: cardiovascular diseases; **HDL-C:** high-density lipoprotein cholesterol; **FH-CVD**: family history of CVD; **MetS:** metabolic syndrome; **SD**: standard deviation; **IQR**: interquartile range | | | |
