# Supplementary material for: Gender differences in changes in metabolic syndrome status and its components and risk of cardiovascular disease: a longitudinal cohort study
Source: Cardiovasc Diabetol. 2022 Nov 2;21:227. doi: 10.1186/s12933-022-01665-8 (PMC9632145; doi:10.1186/s12933-022-01665-8)
Supplement: Supplementary file 3 — Supplementary Material 3 Table S3. Baseline characteristics of the participants and non-participant, Tehran Lipid and Glucose Study [file 12933_2022_1665_MOESM3_ESM.docx]

| **Table S3.** Baseline characteristics of the participants and non-participant, Tehran Lipid and Glucose Study | | | |
| --- | --- | --- | --- |
|  | **Non-participants**  **(n=2017)** | **Participants**  **(n=4624)** | **P-value** |
| **Continuous variable** |  |  |  |
| Age, year | 49.8 (14.1) | 47.9 (12.1) | <0.001 |
| BMI, kg/m^2^ | 27.8 (5.0) | 28.1 (4.5) | 0.039 |
| WC, cm | 94.0 (12.0) | 93.4 (11.2) | 0.124 |
| SBP, mmHg | 120.4 (20.2) | 118.2 (18.5) | <0.001 |
| DBP, mmHg | 75.9 (10.8) | 75.9 (10.4) | 0.998 |
| TG, mmol/L^*^ | 1.6 (1.1) | 1.6 (1.1) | 0.213 |
| FPG, mmol/L^*^ | 5.0 (0.9) | 5.0 (0.8) | 0.137 |
| HDL-C, mmol/L | 1.01 (0.26) | 0.99 (0.26) | 0.129 |
|  |  |  |  |
| **Categorical variable** |  |  |  |
| Smoking |  |  |  |
| Current smoker | 415 (22.1) | 755 (16.4) | <0.001 |
| Past smoker | 189 (10.1) | 388 (8.4) |  |
| never smoker | 1275 (67.9) | 3468 (75.2) |  |
| Education |  |  |  |
| < 6 years | 757 (37.6) | 1570 (34.0) | 0.006 |
| 6-12 years | 967 (48.1) | 2410 (52.2) |  |
| > 12 years | 288 (14.3) | 640 (13.9) |  |
| Marital status |  |  |  |
| Single | 105 (5.2) | 193 (4.2) | 0.002 |
| Married | 1704 (84.6) | 4058 (87.8) |  |
| widowed/divorced | 206 (10.2) | 372 (8.0) |  |
| Physical activity level (low) | 723 (40.2) | 1659 (36.4) | 0.006 |
| FH-CVD (yes) | 213 (10.6) | 452 (9.8) | 0.328 |
| Anti-hypertensive drug use (yes) | 219 (10.9) | 378 (8.2) | 0.001 |
| Anti-diabetic drug use (yes) | 160 (7.9) | 238 (5.1) | <0.001 |
| Lipid-lowering drug use (yes) | 76 (3.8) | 165 (3.6) | 0.721 |
| The characteristics are presented at phase 3 (defined as baseline).  Data are shown as mean (SD) for continuous variables or number (percent) for categorical variables.  * Data are shown as median (IQR), due to skewed distribution, and comparisons were done by Mann–Whitney U test.  **SBP**: systolic blood pressure; **DBP**: diastolic blood pressure; **BMI**: body mass index; **FPG**: fasting plasma glucose; **TG**: Triglycerides; **CVD**: cardiovascular diseases; **HDL-C:** high-density lipoprotein cholesterol; **FH-CVD**: family history of CVD; **MetS:** metabolic syndrome; **SD**: standard deviation; **IQR**: interquartile range | | | |
