# Supplementary material for: Gender differences in changes in metabolic syndrome status and its components and risk of cardiovascular disease: a longitudinal cohort study
Source: Cardiovasc Diabetol. 2022 Nov 2;21:227. doi: 10.1186/s12933-022-01665-8 (PMC9632145; doi:10.1186/s12933-022-01665-8)
Supplement: Supplementary file 4 — Supplementary Material 4 Table S4: Study population stratified by the metabolic syndrome status at Phase 2 and Phase 3 [file 12933_2022_1665_MOESM4_ESM.docx]

| **Table S4:** Study population stratified by the metabolic syndrome status at Phase 2 and Phase 3 | | | | | |
| --- | --- | --- | --- | --- | --- |
|  |  | **Mets status** | | | |
|  |  | **Phase 2** | **n (%)** | **Phase 3** | **n (%)** |
| **Women** | **MetS status** | **Yes** | 1172 (43.6) | **Yes** | 884 (75.4) |
|  |  |  |  | **No** | 288 (24.6) |
|  |  | **No** | 1512 (56.4) | **Yes** | 210 (13.9) |
|  |  |  |  | **No** | 1302 (86.1) |
|  | | | | | |
| **Men** | **MetS status** | **Yes** | 921 (47.5) | **Yes** | 684 (74.3) |
|  |  |  |  | **No** | 237 (25.7) |
|  |  | **No** | 1019 (52.5) | **Yes** | 215 (21.1) |
|  |  |  |  | **No** | 804 (78.9) |
| Values display the number (percent)  **MetS:** metabolic syndrome; **Phase 2:** 2002-2005; **Phase 3**: 2005-2008 | | | | | |
