# Supplementary material for: Gender differences in changes in metabolic syndrome status and its components and risk of cardiovascular disease: a longitudinal cohort study
Source: Cardiovasc Diabetol. 2022 Nov 2;21:227. doi: 10.1186/s12933-022-01665-8 (PMC9632145; doi:10.1186/s12933-022-01665-8)
Supplement: Supplementary file 5 — Supplementary Material 5 Table S5: Incidence rates for CVD and CHD according to the baseline MetS* and change in MetS status, Tehran Lipid and Glucose Study [file 12933_2022_1665_MOESM5_ESM.docx]

| **Table S5:** Incidence rates for CVD and CHD according to the baseline MetS* and change in MetS status, Tehran Lipid and Glucose Study | | | | | | | |
| --- | --- | --- | --- | --- | --- | --- | --- |
|  | **Women (n=2684)** | | |  | **Men (n=1940)** | | |
|  | **Events/ n** | **Person-Years** | **Incidence rate per 1000 person-years** |  | **Events/ n** | **Person-Years** | **Incidence rate per 1000 person-years** |
| **CVD** |  |  |  |  |  |  |  |
| **MetS at baseline** |  |  |  |  |  |  |  |
| No | 84/1590 | 26832.8 | 3.1 (2.5-3.8) |  | 134/1041 | 16741.8 | 8.0 (6.7-9.4) |
| yes | 208/1094 | 17480.5 | 11.8 (10.3-13.6) |  | 193/899 | 13994.5 | 13.7 (11.9-15.8) |
|  |  |  |  |  |  |  |  |
| **Change in MetS** |  |  |  |  |  |  |  |
| MetS-free | 55/1302 | 14591.2 | 3.7 (2.8-4.9) |  | 101/804 | 8488.8 | 11.8 (9.714.4) |
| MetS-recovery | 29/288 | 3103.6 | 9.3 (6.4-13.4) |  | 33/237 | 2448.1 | 13.4 (9.5-18.9) |
| MetS-developed | 24/210 | 2220.9 | 10.8 (7.2-16.1) |  | 31/215 | 2189.7 | 14.1 (9.9-20.1) |
| MetS-stable | 184/884 | 8881.0 | 20.7 (17.9-23.9) |  | 162/684 | 6688.9 | 24.2 (20.7-28.2) |
|  |  |  |  |  |  |  |  |
|  |  |  |  |  |  |  |  |
| **CHD** |  |  |  |  |  |  |  |
| **MetS at baseline** |  |  |  |  |  |  |  |
| No | 65/1590 | 26884.1 | 2.4 (1.8-3.0) |  | 117/1041 | 16794.2 | 6.9 (5.8-8.3) |
| yes | 165/1094 | 17615.5 | 9.3 (8.0-10.9) |  | 165/899 | 14091.9 | 11.7 (10.0-13.6) |
|  |  |  |  |  |  |  |  |
| **Change in MetS** |  |  |  |  |  |  |  |
| MetS-free | 45/1302 | 14616.9 | 3.1 (2.2-4.1) |  | 87/804 | 8530.5 | 10.2 (8.2-12.5) |
| MetS-recovery | 20/288 | 3129.1 | 12.2 (8.5-17-4) |  | 30/237 | 2458.7 | 12.2 (8.5-17.4) |
| MetS-developed | 18/210 | 2243.7 | 8.0 (5.0-12.7) |  | 25/215 | 2206.5 | 11.3 (7.6-16.7) |
| MetS-stable | 147/884 | 8993.3 | 16.3 (13.9-19.2) |  | 140/684 | 6769.5 | 20.6 (17.5-24.4) |
| **MetS:** metabolic syndrome; **CI**: confidence interval; **CVD**: cardiovascular disease; **CHD**: coronary heart disease  * Baseline was defined as Phase 3 (2005-2008) | | | | | | | |
