# Supplementary material for: Gender differences in changes in metabolic syndrome status and its components and risk of cardiovascular disease: a longitudinal cohort study
Source: Cardiovasc Diabetol. 2022 Nov 2;21:227. doi: 10.1186/s12933-022-01665-8 (PMC9632145; doi:10.1186/s12933-022-01665-8)
Supplement: Supplementary file 6 — Supplementary Material 6 Figure S1: Association of MetS at baseline with the risk of CVD (left) and CHD (right), Tehran Lipid and Glucose Study. HR: hazard ratio; MetS: metabolic syndrome; CVD: Cardiovascular disease; CHD: Coronary heart disease. HR was estimated using COX regression model adjusted for age, smoking status, physical activity level, education, marital status, family history of CVD, body mass index [file 12933_2022_1665_MOESM6_ESM.docx]

| 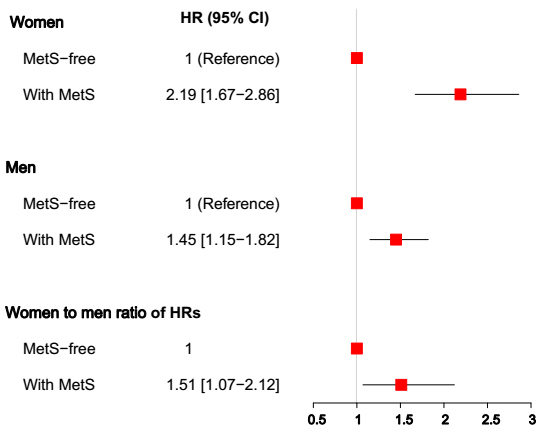 | 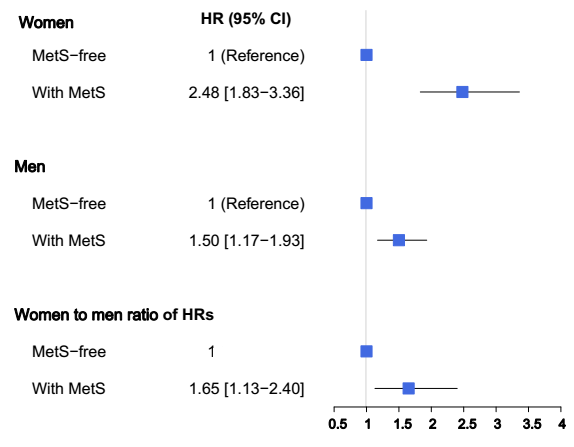 |
| --- | --- |
| **CVD** | **CHD** |
| **Figure S1:** Association of MetS at baseline with the risk of CVD (left) and CHD (right), Tehran Lipid and Glucose Study  **HR**: hazard ratio; **MetS:** metabolic syndrome; **CVD**: Cardiovascular disease; **CHD:** Coronary heart disease  HR was estimated using COX regression model adjusted for age, smoking status, physical activity level, education, marital status, family history of CVD, body mass index | |
