# Supplementary material for: Gender differences in changes in metabolic syndrome status and its components and risk of cardiovascular disease: a longitudinal cohort study
Source: Cardiovasc Diabetol. 2022 Nov 2;21:227. doi: 10.1186/s12933-022-01665-8 (PMC9632145; doi:10.1186/s12933-022-01665-8)
Supplement: Supplementary file 7 — Supplementary Material 7 Table S6: Association of MetS components at *baseline with the risk of CVD, Tehran Lipid and Glucose Study [file 12933_2022_1665_MOESM7_ESM.docx]

| **Table S6:** Association of MetS components at *baseline with the risk of CVD, Tehran Lipid and Glucose Study | | | | | | | | | | |
| --- | --- | --- | --- | --- | --- | --- | --- | --- | --- | --- |
|  | **Women (n=2684)** | | | |  | **Men (n=1940)** | | | |  |
| **Parameter** |  |  | **Model 1** | **Model 2** |  |  |  | **Model 1** | **Model 2** |  |
|  | **Events/n** | **Incidence Rate per 1000 Person-Years** | **HR (95% CI)** | **HR (95% CI)** |  | **Events/n** | **Incidence Rate per 1000 Person-Years** | **HR (95% CI)** | **HR (95% CI)** | *** Women-to-men RHR** |
| **High WC** |  |  |  |  |  |  |  |  |  |  |
| No | 108/1512 | 4.2 (3.5-5.1) | Reference | Reference |  | 118/811 | 9.1 (7.6-11.0) | Reference | Reference | - |
| Yes | 184/1172 | 9.6 (8.3-11.1) | 1.55 (1.22-1.97) | 1.21 (0.91-1.60) |  | 209/1129 | 11.6 (10.1-13.3) | 1.19 (0.95-1.49) | 0.97 (0.75-1.26) | 1.24 (0.89-1.73) |
| **High FPG** |  |  |  |  |  |  |  |  |  |  |
| No | 142/1962 | 4.3 (3.6-50.9) | Reference | Reference |  | 200/1427 | 8.7 (7.5-10.0) | Reference | Reference | - |
| Yes | 150/722 | 13.0 (11.1-15.3) | 2.15 (1.70-2.71) | 1.77 (1.39-2.25) |  | 127/513 | 16.3 (13.7-19.4) | 1.46 (1.17-1.83) | 1.35 (1.07-1.69) | 1.31 (0.95-1.81) |
| **High TG** |  |  |  |  |  |  |  |  |  |  |
| No | 101/1395 | 4.3 (3.5-5.2) | Reference | Reference |  | 151/950 | 10.0 (8.5-11.7) | Reference | Reference | - |
| Yes | 191/1289 | 9.1 (7.9-10.5) | 1.59 (1.25-2.03) | 1.29 (1.00-1.66) |  | 176/990 | 11.2 (9.6-13.0) | 1.27 (1.02-1.57) | 1.10 (0.87-1.38) | 1.17 (0.84-1.62) |
| **Low HDL-C** |  |  |  |  |  |  |  |  |  |  |
| No | 70/704 | 5.9 (4.7-7.5) | Reference | Reference |  | 101/670 | 9.4 (7.8-11.5) | Reference | Reference | - |
| Yes | 222/1980 | 6.8 (5.9-7.7) | 1.14 (0.87-1.49) | 0.95 (0.72-1.26) |  | 226/1270 | 11.2 (9.8-12.8) | 1.34 (1.06-1.70) | 1.13 (0.88-1.44) | 0.84 (0.59-1.21) |
| **High BP** |  |  |  |  |  |  |  |  |  |  |
| No | 115/1906 | 3.5 (2.9-4.3) | Reference | Reference |  | 156/1278 | 7.5 (6.4-8.8) | Reference | Reference | - |
| Yes | 177/778 | 14.4 (12.4-16.7) | 2.37 (1.85-3.02) | 1.97 (1.53-2.53) |  | 171/662 | 16.9 (14.6-19.7) | 1.40 (1.12-1.76) | 1.38 (1.10-1.74) | 1.42 (1.03-1.97) |
| **Model 1:** Adjusted for age **Model 2:** Adjusted for age, smoking status, physical activity level, education, marital status, family history of CVD, body mass index + other components of MetS.  ***Women to men RHR:** The value shows women-to-men relative hazard ratio for each parameter obtained in model 2 adjusted for age, smoking status, physical activity level, education, marital status, family history of CVD, body mass index + other components of MetS.  **MetS**: metabolic syndrome; **CVD**: cardiovascular diseases; **RHR:** ratio of hazard ratios; **BP**: blood pressure; **FPG**: fasting plasma glucose; **TG**: Triglycerides; **HDL-C:** high-density lipoprotein cholesterol; **WC**; waist circumference  * Baseline was defined as Phase 3 (2005-2008) | | | | | | | | | | |
