# Supplementary material for: Gender differences in changes in metabolic syndrome status and its components and risk of cardiovascular disease: a longitudinal cohort study
Source: Cardiovasc Diabetol. 2022 Nov 2;21:227. doi: 10.1186/s12933-022-01665-8 (PMC9632145; doi:10.1186/s12933-022-01665-8)
Supplement: Supplementary file 8 — Supplementary Material 8 Table S7: Association of MetS components at *baseline with the risk of CHD, Tehran Lipid and Glucose Study [file 12933_2022_1665_MOESM8_ESM.docx]

| **Table S7:** Association of MetS components at *baseline with the risk of CHD, Tehran Lipid and Glucose Study | | | | | | | | | | |
| --- | --- | --- | --- | --- | --- | --- | --- | --- | --- | --- |
|  | **Women (n=2684)** | | | |  | **Men (n=1940)** | | | |  |
|  |  |  | **Model 1** | **Model 2** |  |  |  | **Model 1** | **Model 2** |  |
| **Parameter** | **Events/n** | **Incidence Rate per 1000 Person-Years** | **HR (95% CI)** | **HR (95% CI)** |  | **Events/n** | **Incidence Rate per 1000 Person-Years** | **HR (95% CI)** | **HR (95% CI)** | *** Women-to-men RHR** |
| **High WC** |  |  |  |  |  |  |  |  |  |  |
| No | 86/1512 | 3.3 (2.7-4.1) | Reference | Reference |  | 102/811 | 7.9 (6.5-9.6) | Reference | Reference | - |
| Yes | 144/1172 | 7.5 (3.7-8.8) | 1.58 (1.20-2.07) | 1.33 (0.97-1.83) |  | 180/1129 | 9.9 (8.6-11.5) | 1.19 (0.94-1.52) | 1.04 (0.78-1.38) | 1.28 (0.89-1.85) |
| **High FPG** |  |  |  |  |  |  |  |  |  |  |
| No | 105/513 | 3.1 (2.6-3.8) | Reference | Reference |  | 177/1427 | 7.6 (6.6-8.9) | Reference | Reference | - |
| Yes | 125/722 | 10.8 (9.1-12.9) | 2.52 (1.94-3.28) | 2.09 (1.60-2.74) |  | 105/513 | 13.3 (11.0-16.1) | 1.39 (1.09-1.77) | 1.28 (1.01-1.64) | 1.63 (1.14-2.33) |
| **High TG** |  |  |  |  |  |  |  |  |  |  |
| No | 74/1395 | 3.1 (2.5-3.9) | Reference | Reference |  | 124/950 | 8.1 (6.8-9.7) | Reference | Reference | - |
| Yes | 156/1289 | 7.4 (6.3-8.6) | 1.83 (1.39-2.42) | 1.45 (1.09-1.94) |  | 158/990 | 10.0 (8.5-11.7) | 1.37 (1.08-1.73) | 1.17 (0.91-1.50) | 1.23 (0.85-1.78) |
| **Low HDL-C** |  |  |  |  |  |  |  |  |  |  |
| No | 49/704 | 4.1 (3.1-5.5) | Reference | Reference |  | 82/670 | 7.6 (6.1-9.5) | Reference | Reference | - |
| Yes | 181/1980 | 5.5 (4.7-6.3) | 1.33 (0.97-1.83) | 1.08 (0.78-1.49) |  | 200/1270 | 9.9 (8.6-11.3) | 1.44 (1.11-1.86) | 1.18 (0.90-1.55) | 0.91 (0.60-1.37) |
| **High BP** |  |  |  |  |  |  |  |  |  |  |
| No | 95/1906 | 2.9 (2.4-3.6) | Reference | Reference |  | 141/1278 | 6.8 (5.7-8.0) | Reference | Reference | - |
| Yes | 135/778 | 10.9 (9.2-12.9) | 2.31 (1.76-3.04) | 1.91 (1.45-2.53) |  | 141/662 | 13.8 (11.7-16.3) | 1.34 (1.05-1.71) | 1.34 (1.05-1.72) | 1.42 (1.01-2.04) |
| **Model 1:** Adjusted for age **Model 2:** Adjusted for age, smoking status, physical activity level, education, marital status, family history of CVD, body mass index + other components of MetS.  ***Women to men RHR:** The value shows women-to-men relative hazard ratio for each parameter obtained in model 2 adjusted for age, smoking status, physical activity level, education, marital status, family history of CVD, body mass index + other components of MetS.  **MetS**: metabolic syndrome; **CVD**: cardiovascular diseases; **RHR:** ratio of hazard ratios; **BP**: blood pressure; **FPG**: fasting plasma glucose; **TG**: Triglycerides; **HDL-C:** high-density lipoprotein cholesterol; **WC**; waist circumference  * Baseline was defined as Phase 3 (2005-2008) | | | | | | | | | | |
